# Network pharmacology-based strategy to investigate the bioactive ingredients and molecular mechanism of *Evodia rutaecarpa* in colorectal cancer

**DOI:** 10.1186/s12906-023-04254-8

**Published:** 2023-12-01

**Authors:** Yongqu Lu, Kangdi Dong, Meng Yang, Jun Liu

**Affiliations:** 1 Department of Breast and Thyroid Surgery, China-Japan Friendship Hospital, Beijing, 100029 China; 2 Department of Gastrointestinal Surgery, Shandong Provincial Hospital Affiliated to Shandong First Medical University, Jinan, 250021 China

**Keywords:** Colorectal cancer, Evodia rutaecarpa, Network pharmacology, Rutaecarpine, Tumor necrosis factor

## Abstract

**Background:**

*Evodia rutaecarpa*, a traditional herbal drug, is widely used as an analgesic and antiemetic. Many studies have confirmed that *Evodia rutaecarpa* has an anticancer effect. Here, our study explored the bioactive ingredients in *Evodia rutaecarpa* acting on colorectal cancer (CRC) by utilizing network pharmacology.

**Methods:**

We clarified the effective ingredients and corresponding targets of *Evodia rutaecarpa*. CRC-related genes were obtained from several public databases to extract candidate targets. Candidate targets were used to construct a protein–protein interaction (PPI) network for screening out core targets with topological analysis, and then we selected the core targets and corresponding ingredients for molecular docking. Cell proliferation experiments and enzyme-linked immunosorbent assays (ELISAs) verified the anticancer effect of the bioactive ingredients and the results of molecular docking.

**Results:**

Our study obtained a total of 24 bioactive ingredients and 100 candidate targets after intersecting ingredient-related targets and CRC-related genes, and finally, 10 genes—*TNF*, *MAPK1*, *TP53*, *AKT1*, *RELA*, *RB1*, *ESR1*, *JUN*, *CCND1* and *MYC*—were screened out as core targets. In vitro experiments suggested that rutaecarpine excelled isorhamnetin, evodiamine and quercetin in the inhibition of CRC cells and the release of TNF-α was altered with the concentrations of rutaecarpine. Molecular docking showed that rutaecarpine could effectively bind with TNF-α.

**Conclusion:**

The pairs of ingredients-targets in *Evodia rutaecarpa* acted on CRC were excavated. Rutaecarpine as a bioactive ingredient of *Evodia rutaecarpa*might effectively inhibit the proliferation of CRC cells by suppressing TNF-α.

**Supplementary Information:**

The online version contains supplementary material available at 10.1186/s12906-023-04254-8.

## Background

Colorectal cancer (CRC) is a frequently occurring malignancy originating from the digestive tract with high invasiveness. In terms of global incidence, CRC ranks third in terms of cancer-related mortality [1]. Advanced explorations of tumorigenesis and pathophysiology have expanded the available tools for preventing and treating CRC, leading to individual treatment plans [2]. As CRC is a complicated malignant disease affected by many factors, its pathogenesis and development remain diversified [3]. Appropriate drugs can relieve the progression of CRC to some extent, especially for patients not eligible for non-radical excision, but anticancer medicine is not broadly efficacious and comes with potential side effects [4]. For many years 5-fluorouracil (5-FU) was the only chemotherapeutic agent available to improve survival in CRC patients but the treatment for metastatic CRC had been considered palliative. Different strategies of therapy with anti-vascular endothelial growth factor, anti-epidermal growth factor receptor or traditional Chinese medicine significantly improved prognosis and relieved cumulative toxicities [5, 6]. The sources of anticancer drugs are various with emerging studies [7].

Herbal drugs are a critical part of traditional Chinese medicine. However, as a result of the complexity of the components, some side effects, including organ failure, accompany the significant efficacy of these drugs against diseases [8]. Many biologically active constituents have been isolated from herbal drugs and confirmed to inhibit tumour growth in experimental and clinical studies [9]. *Evodia rutaecarpa* (Chinese name: Wuzhuyu), a traditional herbal drug, has been widely used to treat headaches and abdominal pain for a long time [10]. As a natural product with complex components, *Evodia rutaecarpa* has been shown to be involved in the biological regulation of tumour growth [11]. Evodiamine from *Evodia rutaecarpa* was confirmed to induce apoptosis via activation of the endoplasmic reticulum stress signalling pathway in ovarian cancer [12]. Rutaecarpine, another constituent of *Evodia rutaecarpa*, could overcome castration resistance to anti-androgen therapy in prostate cancer and significantly improve dextran sulfate sodium-induced colitis [13, 14]. The active ingredients in *Evodia rutaecarpa* therefore might be potential tools for anticancer therapy.

Network pharmacology was developed based on the theory that compounds that selectively act on two or more targets should be more effective than agents that act on a single target [15]. Mapping the network of potential interactions between agents and targets is being gradually applied for drug discovery with the goal of exploring target molecules, side effects and drug resistance to conceive a novel treatment [16]. Safer and more effective drug selection requires the design of ligands with published structures in pharmaceutical datasets or chemistry literature and the characterization of their targets [17]. Another advantage is that network pharmacology is much more efficient than traditional drug discovery approaches in terms of the development of successful drugs [18].

The effects of *Evodia rutaecarpa* on CRC can be integrated with network pharmacology to map the unexplored target space and confirm the therapeutic potential. We focused on the functions and mechanisms of *Evodia rutaecarpa* in CRC. Network pharmacology was applied and revealed the ingredients and corresponding targets that were affected in CRC, and we selected pairs with strong correlations and prominent impacts for further validation, which included computational analysis and experimental verification. Our study indicates the anti-cancer application of rutaecarpine and expands the knowledge regarding promising therapeutics from *Evodia rutaecarpa* for the treatment of CRC.

## Methods

### Screening of bioactive ingredients and targets in *Evodia rutaecarpa*

The bioactive ingredients of *Evodia rutaecarpa* were searched on the Traditional Chinese Medicine Database and Analysis Platform (TCMSP, https://tcmsp-e.com) [19]. Oral bioavailability (OB) refers to the rate and extent of drug absorption and its availability at the site of action. Drug-likeness (DL) reflects the similarity of the nature of drugs that possess specific molecular properties. We screened the final bioactive ingredients with the criteria of OB > 30% and DL > 0.18 supported by the previous literature [20]. Corresponding targets of the included ingredients were also downloaded from TCMSP and converted to normative gene names with a protein sequence and annotation in The Universal Protein Resource (UniProt, https://www.UniProt.org) [21].

### Screening of candidate targets for CRC

CRC-related targets of *Homo sapiens* were collected from public databases, including GeneCards (https://www.genecards.org), Online Mendelian Inheritance in Man (OMIM, https://omim.org), Pharmacogenomics Knowledgebase (PharmGKB, https://www.pharmgkb.org), Therapeutic Target Database (TTD, http://db.idrblab.net/ttd) and DrugBank (https://go.drugbank.com) [22–26]. The targets from GeneCards with relevance scores > 10 were selected. All CRC-related targets were gathered and intersected with ingredient-related targets to identify candidate targets for further analysis. The network of overlapping targets and corresponding bioactive ingredients was visualized using Cytoscape software (version 3.7.1).

### Functional enrichment analyses of candidate targets

Functional enrichment analyses were performed in R software (version 3.6.0). Gene ontology (GO) analysis and Kyoto Encyclopedia of Genes and Genomes (KEGG) analysis were conducted to investigate the biological functions and pathways related to the candidate targets with the clusterProfiler package [27].

### Interaction network construction and core targets extraction

The network platform STRING (https://string-db.org) was used to construct the protein–protein interaction (PPI) network. Candidate targets were uploaded to STRING with the organism *Homo sapiens*. The minimum required interaction score was set as the highest confidence (0.9), and disconnected nodes were removed from the network.

The CytoNCA plugin for Cytoscape software was applied for topological analysis to identify central nodes based on the indicators of betweenness, closeness, degree, eigenvector, local average connectivity-based method and network. The core targets extracted from the network met the requirement that all indicators of targets need to be greater than the medians of the corresponding sets.

### Molecular docking

The structures of the drug molecule and target macromolecule were downloaded from PubChem (https://pubchem.ncbi.nlm.nih.gov) and The Protein Data Bank (PDB, https://www.rcsb.org) [28, 29], respectively. The hydration and existing ligand of the receptor were removed using PyMOL software (version 2.4.1). AutoDockTools software (version 1.5.6) was applied for format conversion and location of the active pocket. We used AutoDock Vina for docking exploration with optional combinations [30]. The resulting molecular docking was visualized with PyMOL software.

### Cell culture

The human CRC cell lines HT29 and LS180 were obtained from Cell Resource Center, Institute of Basic Medical Sciences, Chinese Academy of Medical Sciences. The cells were routinely grown in Dulbecco’s modified Eagle’s medium (DMEM, Corning, USA) containing 10% foetal bovine serum (Gibco, USA) and 1% penicillin-streptomycin solution (HyClone, USA) in an incubator with a humidified atmosphere of 5% CO_2_ at 37 °C.

### Cell proliferation assay

Cells (8 × 10^3^ per well) were seeded in 96-well plates. After attachment to the plates and culture in complete growth medium for 24 h, the cells were treated with isorhamnetin, evodiamine, quercetin, rutaecarpine (MedChemExpress, China) containing the concentrations of 0.5 µM, 1 µM, 2 µM, 4 µM, 8 µM and 16 µM.0.1% dimethyl sulfoxide (DMSO, MP Biomedicals, USA) was used as a solvent control. Cell Counting Kit-8 (CCK-8) reagent (APExBIO, USA) was added 48 h later, and the optical density (OD) value per well at 450 nm was detected with a Thermo Varioskan Flash reader after incubation for 2 h. The experiment was repeated three times. The relative viability was calculated as (treated sample OD-blank control OD)/ (negative control OD-blank control OD) *100.

### Enzyme-linked immunosorbent assay (ELISA)

CRC cells (2.4 × 10^5^ per well) were seeded in 6-well plates and grown in 2 mL complete medium for 24 h before treatment with 0.5 µM, 1 µM, 2 µM and 4 µM rutaecarpine for 48 h. The culture supernatant was collected for ELISA (Jianglai Biological, China) following the manufacturers’ protocol.

### Statistical analysis

All statistical analyses were performed using SPSS software (version 27). Data are shown as means ± standard deviation (SD). Statistical differences were analysed by one-way analysis of variance (ANOVA) followed by Fisher’s least significant difference (LSD) and t-test. A *P*-value < 0.05 was considered statistically significant. The *Q*-value was calculated according to the adjusted *P*-value and the false discovery rate.

## Results

### Sets of bioactive ingredients and targets in *Evodia rutaecarpa*

The chemical compounds of *Evodia rutaecarpa* are complex and mainly include berberine, rutaecarpine, obacunone, isorhamnetin and other chemical compounds. A total of 30 chemical compounds from *Evodia rutaecarpa* were identified according to the inclusion criteria (Additional file 1). Subsequently, 427 pairs of 24 bioactive ingredients and 180 targets were screened for further analysis (Additional file 2).

### Sets of targets in CRC and selection of candidate targets

**Fig. 1:**
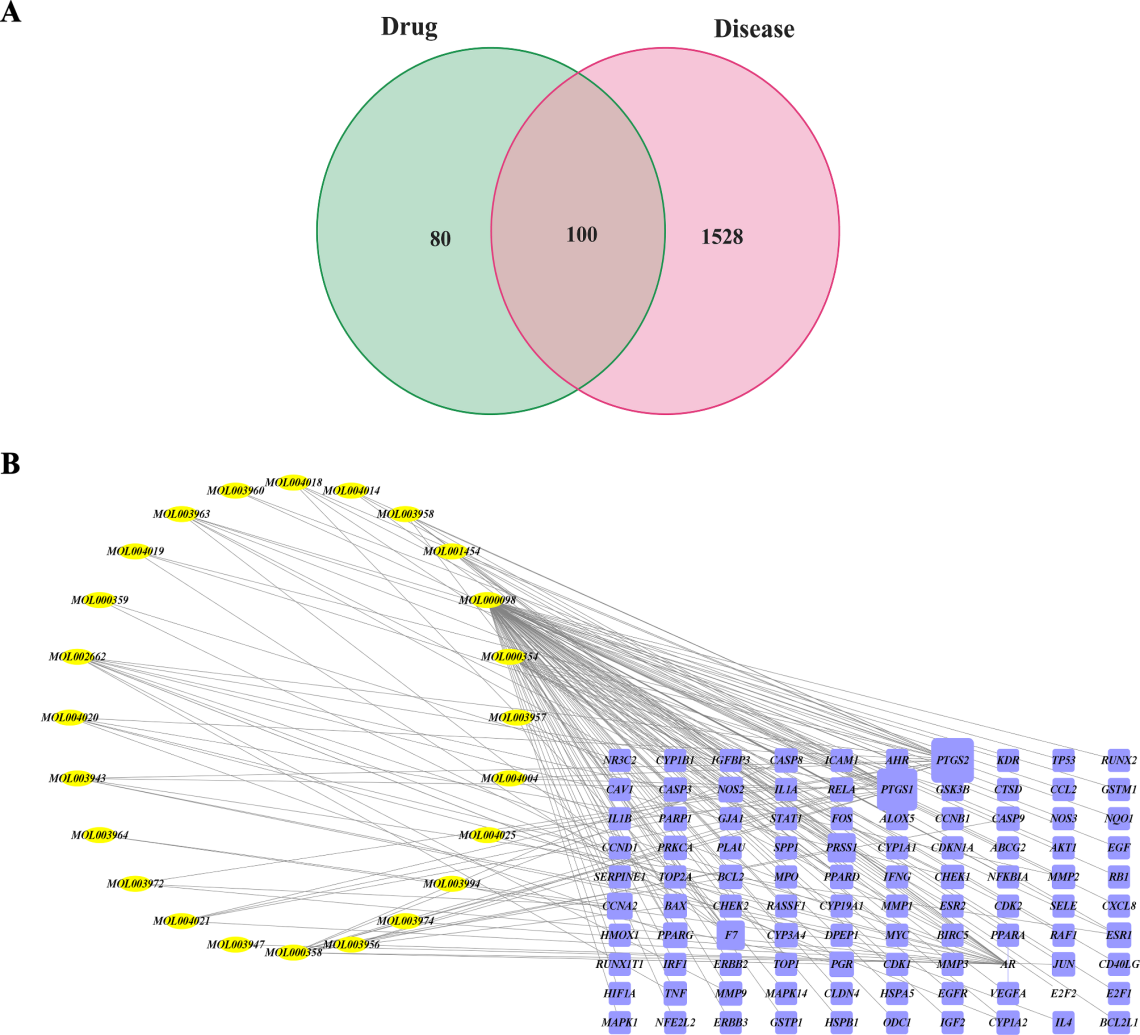
Selection and visualization of candidate targets. (**A**) The candidate targets were selected as the common genes. The green stood for *Evodia rutaecarpa*-related genes and red for CRC-related genes. (**B**) The network of bioactive ingredients and candidate targets of *Evodia rutaecarpa* was visualized by Cytoscape

After deleting duplicates, we obtained 1,628 CRC-related genes, including 36 from DrugBank, 1,261 from GeneCards, 168 from OMIM, 298 from PharmGKB and 90 from TTD. After mapping *Evodia rutaecarpa*-related targets and CRC-related targets, 100 overlapping targets and corresponding target-related bioactive ingredients were identified (Fig. 1A). Finally, the network comprising 24 bioactive ingredients and 100 candidate genes that were indicated targets of *Evodia rutaecarpa* in the treatment of CRC was visualized by Cytoscape (Fig. 1B).

### Functional enrichment analysis of candidate targets

**Fig. 2:**
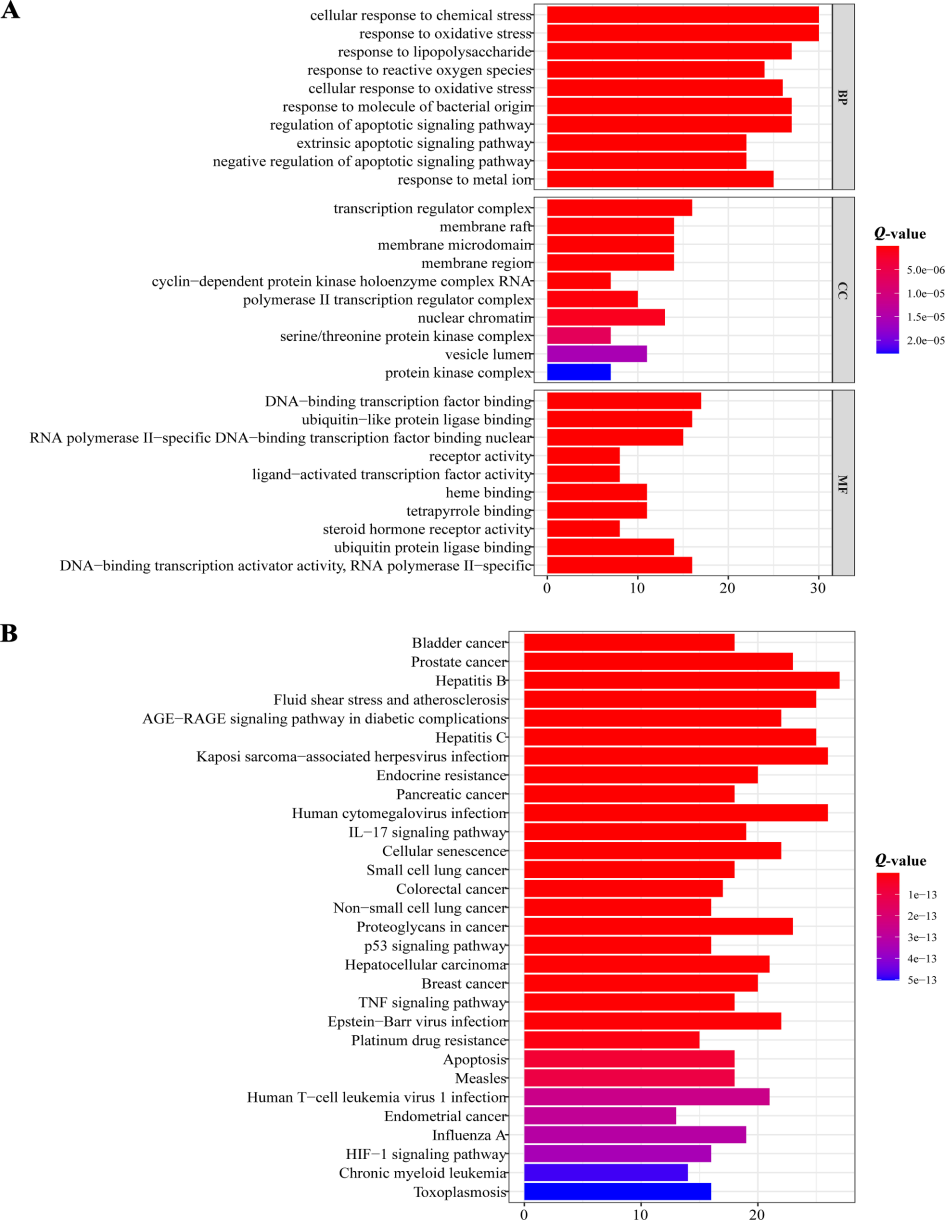
Functional enrichment analysis of candidate targets. (**A**) GO analysis of candidate targets. (**B**) KEGG analysis of candidate targets. BP: biological process. CC: cellular component. MF: molecular function

We performed GO analysis of the candidate targets, and the top three GO terms for biological processes were the cellular response to chemical stress, the response to oxidative stress and the response to lipopolysaccharide, all of which are closely associated with tumorigenesis and tumour progression (Fig. 2A). The majority of the enriched KEGG pathways were related to several types of cancer, including CRC, as expected (Fig. 2B).

### Network construction and core targets screening

**Fig. 3:**
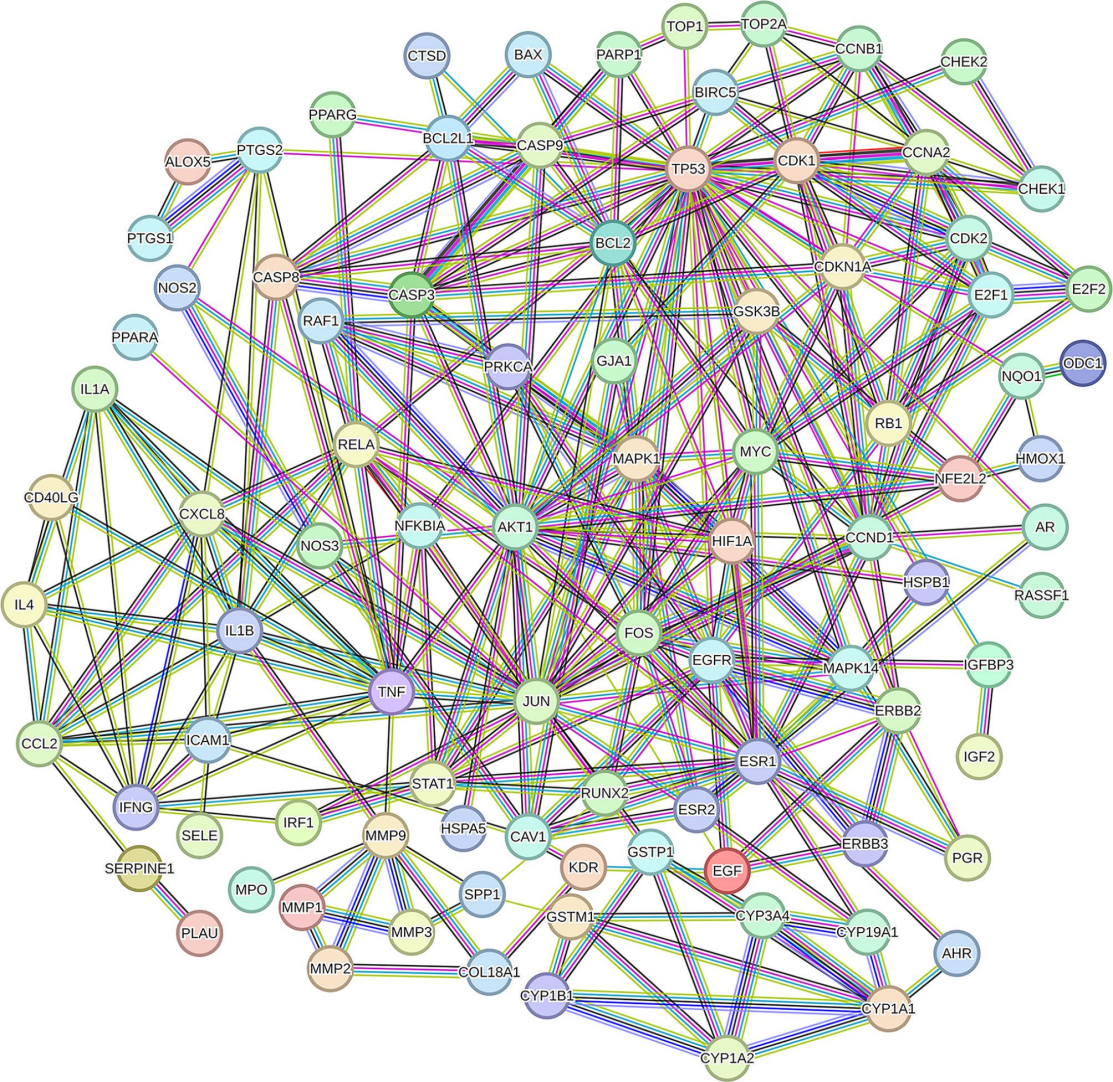
Interaction of candidate targets. PPI network from STRING.

**Fig. 4:**
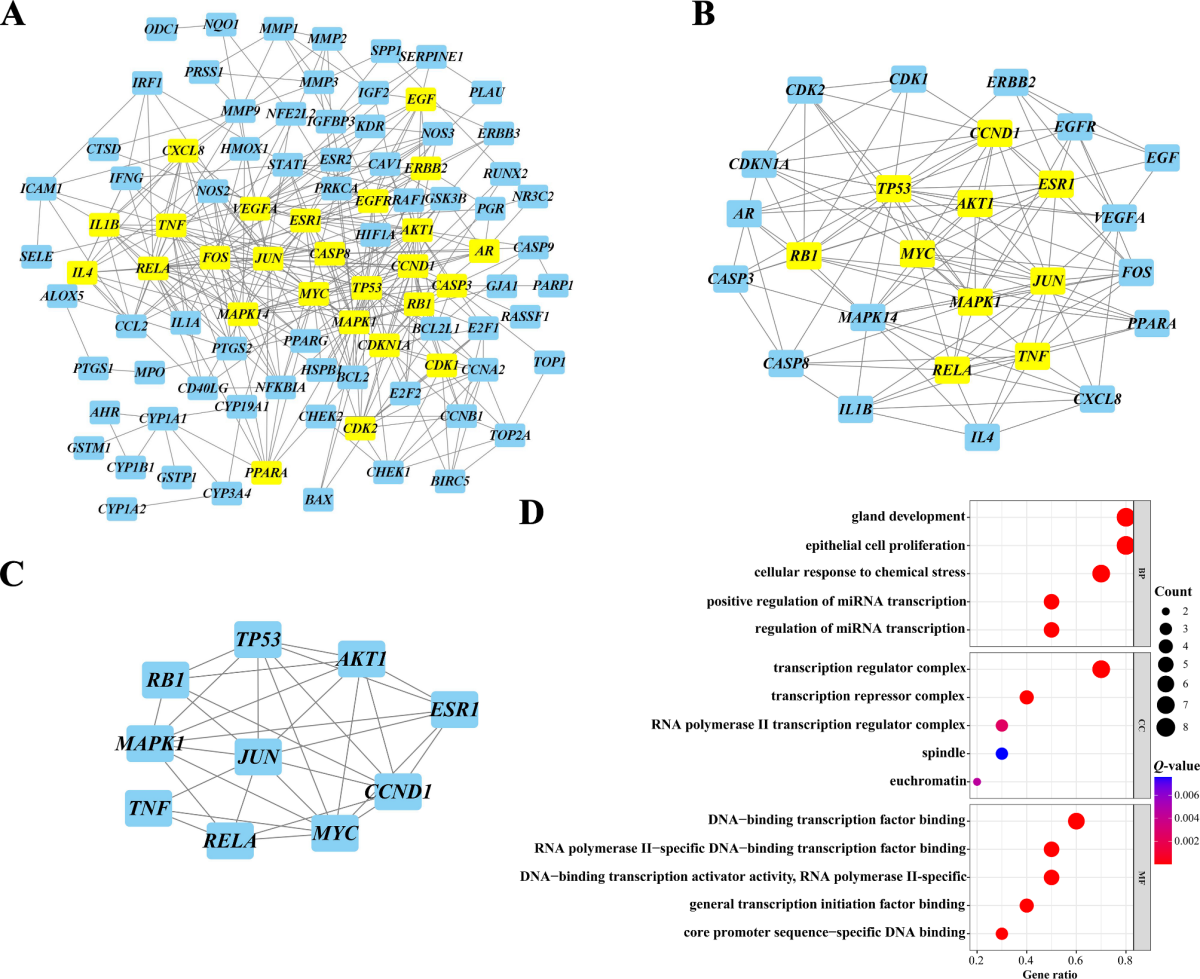
Selection of core targets. Core targets were selected after first (**A**), second (**B**) and third (**C**) topological analysis. (**D**) Final 10 core targets were included for GO analysis. BP: biological process. CC: cellular component. MF: molecular function

To explore the mechanism of *Evodia rutaecarpa* in CRC, a PPI network was built by importing the 100 overlapping targets into the STRING database. The target network had 100 nodes and 380 edges, with an average node degree of 7.6 (Fig. 3). The data for the network constructed in the STRING database were imported into Cytoscape, and the core targets were selected after three topological analyses (Fig. 4A, B&C). The top 10 targets *TNF*, *MAPK1*, *TP53*, *AKT1*, *RELA*, *RB1*, *ESR1*, *JUN*, *CCND1* and *MYC* were considered to play crucial roles in the treatment of CRC. Functional enrichment analysis suggested final 10 targets were associated with DNA-binding transcription and cell proliferation (Fig. 4D).

### Validation with experiments and molecular docking

**Fig. 5:**
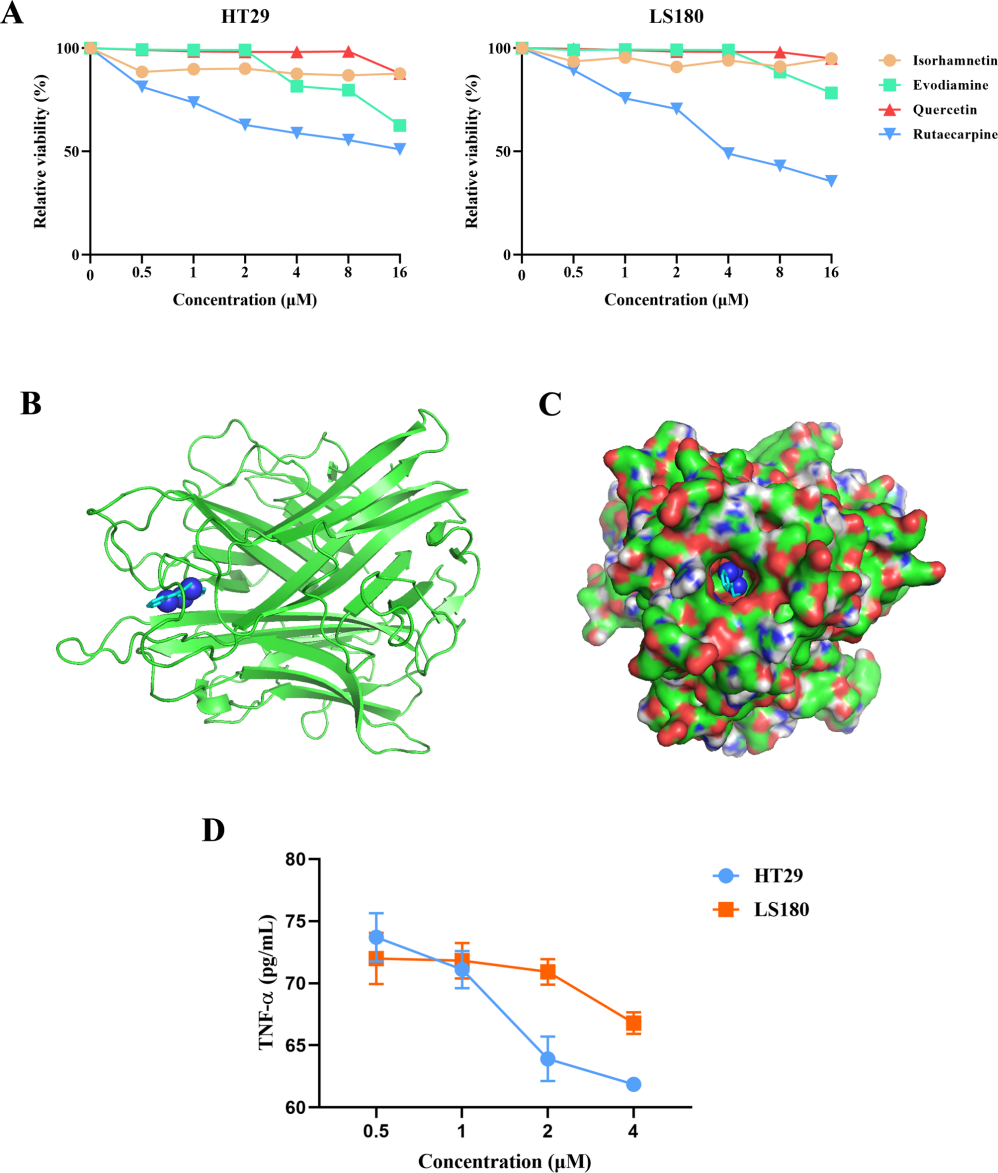
Validation by in vitro experiments and molecular docking. (**A**) The proliferative ability of HT29 and LS180 cells that disposed in different concentrations of isorhamnetin, evodiamine, quercetin and rutaecarpine (0.5 µM, 1 µM, 2 µM, 4 µM, 8 µM and 16 µM). Schematic diagram (**B**) and stereogram (**C**) showed the binding pattern of rutaecarpine and TNF-α in molecular docking. (**D**) The supernatant concentration of TNF-α after HT29 and LS180 cells pretreating with rutaecarpine (0.5 µM, 1 µM, 2 µM and 4 µM). Data was showed as mean or mean ± SD.

The CCK-8 assay was used to assess CRC cell proliferation under main ingredients of *Evodia rutaecarpa*, and the results showed that cell activity was distinctly affected by treatment with rutaecarpine comparing to isorhamnetin, evodiamine and quercetin (Fig. 5A).

Previous study confirmed that rutaecarpine and TNF-α exert activity in colon diseases, and our study suggested TNF-α was proposed to be directly related to *Evodia rutaecarpa* [14, 31]. Based on above evidents, we attempted to use molecular docking forverifying whether rutaecarpine played a significative role in the regulation of TNF-α. The small molecule rutaecarpine showed a compact binding pattern with the active pocket of TNF-α protein with a binding energy value of -9.6 kcal/mol (Fig. 5B&C).

The TNF-α levels were reduced in the culture supernatant after CRC cells were pretreated with decreasing concentrations of rutaecarpine, indicating that rutaecarpine might block the activity of extracellular TNF-α, verifying the conclusion of the molecular docking data (Fig. 5D).

## Discussion

As a traditional herbal drug, *Evodia rutaecarpa* is a prevalent clinical treatment, but the molecular mechanism of its therapeutic function is complex and partially unclear. The effect of the drug was summarized in practical applications, but the molecular mechanism has not been fully clarified. We attempted to identify new indications for established drugs with long-established safety in humans. Thorough research on *Evodia rutaecarpa* revealed valuable candidates. Rutaecarpine, an alkaloid constituent isolated from *Evodia rutaecarpa*, was screened out for further investigation in our study based on the network pharmacology results. It has been reported that rutaecarpine could inhibit angiogenesis as a potential drug candidate [32]. With regard to its anticancer role, rutaecarpine has been reported to protect against reactive oxygen species damage in human hepatic cancer cell lines and against cytotoxicity by activating the Nrf2/ARE pathway [33]. Rutaecarpine administration suppressed the growth of prostate cancer cells subcutaneously injected into mice and restored the immune balance of Th1- and M1-polarized cell types in vivo. Moreover, TNF-α was positively correlated with tumour weight in the experimental mice and considered a potent cancer cachexia factor that could be inhibited by the anti-inflammatory cytokine IL-10 [34]. This finding provided us with a clue for the current investigation that TNF-α might be an anticancer target of rutaecarpine.

TNF-α, a common inflammatory cytokine with important roles in homeostasis and pathogenesis, works as a double-edged sword in cancer behaviour [35]. TNF-α can induce necrosis of cancer cells yet facilitate a malignant phenotype by inducing the synthesis of matrix metalloproteinases [36]. TNF-α inhibitors have already been commercially harnessed and introduced in the clinic with a paradoxical mechanism [37]. As TNF-α is tightly related to clinical characteristics, the expression of TNF-α mRNA extracted from archival tissue specimens in CRC patients clearly showed a strong correlation with advanced disease stage [38]. The function of TNF-α in CRC was also investigated in earlier studies. TNF-α induced invasion and migration of CRC cells depended on the epithelial-mesenchymal transition status and phosphorylation of AKT and glycogen synthase kinase, and one type of herbal prescription could attenuate the related invasive phenotype of TNFα-induced CRC [31]. TNFα is also involved in the regulation of CRC-associated signals and pathways, such as the activation of reactive oxygen species, NF-κB and MAPK, which contribute to angiogenesis in the tumour microenvironment [39]. Anti-TNF drugs such as adalimumab and infliximab have been approved for inflammatory bowel disease in clinic more than two decades [40]. Patients with inflammatory bowel disease with Anti-TNF agents were less likely to develop CRC [41]. The mechanisms of TNF‑α regulated by drug for anti‑inflammatory effects to retard CRC progression have been confirmed in previous studies. Zerumbone, a bioactive ingredient, reduced the proliferation of CRC cells by restraining TNF‑α [42]. Another ingredient evodiamine was proved to inhibited the level of TNF‑α to mediate antitumor effects by in vivo and in vitro CRC experiments [43]. But the clinical applications for CRC still need to be further investigated.

We applied network pharmacology to expand upon the anticancer function of *Evodia rutaecarpa*. We aimed to identify a single ingredient of the herbal drug suitable for unambiguous investigation, and we screened out a few of the major ingredients strongly associated with CRC-related genes in paired form based on data extracted from several public databases. For further confirmation, topological analysis was used to select core genes in the tumorigenesis and progression of CRC as candidate targets. The structures of rutaecarpine and TNF-α were obtained for molecular docking analysis to assess their potential interaction. After this interaction was verified by computational analyses, we performed experimental validation. The anticancer effect of rutaecarpine was verified based on our finding that this molecule could inhibit CRC proliferation. Considering the contradictory functions of TNF-α, we exposed CRC cells to rutaecarpine, and we found that the effective concentrations of TNF-α were significantly upregulated. Thus, rutaecarpine might suppress the malignant behaviour of CRC by inhibiting TNF-α release.

There are some limitations to our study. Firstly, we found a number of significative pairs of ingredients-genes after database analysis and focused on rutaecarpine-TNF-α in our study. The other pairs can be investigated. Secondly, the promotive or inhibitive effects of drugs are often dose-dependent. The regulatory effect of TNF-α on rutaecarpine is still ambiguous, as it has dual roles and the dose may interfere with the final results. Thirdly, more biological experiments including in vivo model and further investigations of the downstream effectors of rutaecarpine for in-depth exploration may better support this function.

## Conclusions

We conducted a systematic investigation of *Evodia rutaecarpa* against CRC with network pharmacology, molecular docking and experiment. Our results indicated that one bioactive ingredient, rutaecarpine, might restrain the biological behaviour of CRC cells via the regulation of TNF-α. Taken together, our work uncovers the effect of rutaecarpine in CRC and contributes to the development of anticancer treatments from traditional herbal drugs.

### Electronic supplementary material

Below is the link to the electronic supplementary material.

Supplementary Material 1

## Data Availability

Publicly available datasets were analyzed in this study. This data can be found here:https://old.tcmsp-e.com/tcmsp.php, https://www.genecards.org, https://omim.org, https://www.pharmgkb.org, http://db.idrblab.net/ttd and https://go.drugbank.com.

## Abbreviations

CRC: colorectal cancer
PPI: protein–protein interaction
ELISA: enzyme-linked immunosorbent assay
5-FU: 5- fluorouracil
TCMSP: Traditional Chinese Medicine Database and Analysis Platform
OB: Oral bioavailability
DL: Drug-likeness
UniProt: Universal Protein Resource
OMIM: Online Mendelian Inheritance in Man
PharmGKB: Pharmacogenomics Knowledgebase
TTD: Therapeutic Target Database
GO: Gene ontology
KEGG: Kyoto Encyclopedia of Genes and Genomes
PDB: Protein Data Bank
DMEM: Dulbecco’s modified Eagle’s medium
DMSO: dimethyl sulfoxide
CCK-8: Cell Counting Kit-8
OD: optical density
SD: standard deviation
LSD: Fisher’s least significant difference
