# Supplementary material for: Network pharmacology-based strategy to investigate the bioactive ingredients and molecular mechanism of Evodia rutaecarpa in colorectal cancer
Source: BMC Complement Med Ther. 2023 Dec 1;23:433. doi: 10.1186/s12906-023-04254-8 (PMC10691004; doi:10.1186/s12906-023-04254-8)
Supplement: Supplementary file 1 — Supplementary Material 1 [file 12906_2023_4254_MOESM1_ESM.docx]

**Additional file 1. Chemical compounds from Evodia rutaecarpa after screening.**

| Molecule Id | Molecule Name | OB (%) | DL |
| --- | --- | --- | --- |
| MOL001454 | berberine | 36.86 | 0.78 |
| MOL002662 | rutaecarpine | 40.3 | 0.6 |
| MOL013352 | Obacunone | 43.29 | 0.77 |
| MOL000354 | isorhamnetin | 49.6 | 0.31 |
| MOL000358 | beta-sitosterol | 36.91 | 0.75 |
| MOL000359 | sitosterol | 36.91 | 0.75 |
| MOL003942 | Rutaevine | 66.05 | 0.58 |
| MOL003943 | Rutalinidine | 40.89 | 0.22 |
| MOL003947 | 1-methyl-2-[(Z)-pentadec-10-enyl]-4-quinolone | 48.45 | 0.46 |
| MOL003950 | 1-methyl-2-[(Z)-undec-6-enyl]-4-quinolone | 48.48 | 0.27 |
| MOL003956 | dihydrorutaecarpine | 42.27 | 0.6 |
| MOL003957 | 1-methyl-2-pentadecyl-4-quinolone | 44.52 | 0.46 |
| MOL003958 | Evodiamine | 86.02 | 0.64 |
| MOL003960 | 1-(5,7,8-trimethoxy-2,2-dimethylchromen-6-yl)ethanone | 30.39 | 0.18 |
| MOL003963 | hydroxyevodiamine | 72.11 | 0.71 |
| MOL003964 | 1-methyl-2-undecyl-4-quinolone | 47.59 | 0.27 |
| MOL003972 | 1-methyl-2-nonyl-4-quinolone | 48.42 | 0.2 |
| MOL003974 | Evocarpine | 48.66 | 0.36 |
| MOL003975 | icosa-11,14,17-trienoic acid methyl ester | 44.81 | 0.23 |
| MOL003994 | 24-methyl-31-norlanost-9(11)-enol | 38 | 0.75 |
| MOL004002 | 5alpha-O-(3'-Methylamino-3'-phenylpropionyl)nicotaxine | 30.86 | 0.49 |
| MOL004004 | 6-OH-Luteolin | 46.93 | 0.28 |
| MOL004014 | Evodiamide | 73.77 | 0.28 |
| MOL004017 | Fordimine | 55.11 | 0.26 |
| MOL004018 | Goshuyuamide I | 83.19 | 0.39 |
| MOL004019 | GoshuyuamideII | 69.11 | 0.43 |
| MOL004020 | gossypetin | 35 | 0.31 |
| MOL004021 | Gravacridoneshlirine | 63.73 | 0.54 |
| MOL004025 | N-(2-Methylaminobenzoyl)tryptamine | 56.96 | 0.26 |
| MOL000098 | quercetin | 46.43 | 0.28 |

**Additional file 2. Preliminary selection of bioactive ingredients and targets.**

| Molecule Id | Molecule Name | Symbol |
| --- | --- | --- |
| MOL001454 | berberine | *NOS2* |
| MOL001454 | berberine | *PTGS1* |
| MOL001454 | berberine | *KCNH2* |
| MOL001454 | berberine | *ESR1* |
| MOL001454 | berberine | *AR* |
| MOL001454 | berberine | *SCN5A* |
| MOL001454 | berberine | *PTGS2* |
| MOL001454 | berberine | *RXRA* |
| MOL001454 | berberine | *ADRB2* |
| MOL001454 | berberine | *HSP90AA2P* |
| MOL001454 | berberine | *PRSS1* |
| MOL001454 | berberine | *NCOA2* |
| MOL001454 | berberine | *PDE10A* |
| MOL001454 | berberine | *CALML3* |
| MOL002662 | rutaecarpine | *PTGS1* |
| MOL002662 | rutaecarpine | *AR* |
| MOL002662 | rutaecarpine | *SCN5A* |
| MOL002662 | rutaecarpine | *PTGS2* |
| MOL002662 | rutaecarpine | *HTR3A* |
| MOL002662 | rutaecarpine | *RXRA* |
| MOL002662 | rutaecarpine | *CHEK1* |
| MOL002662 | rutaecarpine | *MMP2* |
| MOL002662 | rutaecarpine | *MMP9* |
| MOL002662 | rutaecarpine | *TNF* |
| MOL002662 | rutaecarpine | *CYP3A4* |
| MOL002662 | rutaecarpine | *CYP1A2* |
| MOL002662 | rutaecarpine | *IL4* |
| MOL002662 | rutaecarpine | *CYP2B6* |
| MOL000354 | isorhamnetin | *NOS2* |
| MOL000354 | isorhamnetin | *PTGS1* |
| MOL000354 | isorhamnetin | *ESR1* |
| MOL000354 | isorhamnetin | *AR* |
| MOL000354 | isorhamnetin | *PPARG* |
| MOL000354 | isorhamnetin | *PTGS2* |
| MOL000354 | isorhamnetin | *ESR2* |
| MOL000354 | isorhamnetin | *DPP4* |
| MOL000354 | isorhamnetin | *MAPK14* |
| MOL000354 | isorhamnetin | *GSK3B* |
| MOL000354 | isorhamnetin | *HSP90AA2P* |
| MOL000354 | isorhamnetin | *CDK2* |
| MOL000354 | isorhamnetin | *PRSS1* |
| MOL000354 | isorhamnetin | *CCNA2* |
| MOL000354 | isorhamnetin | *NCOA2* |
| MOL000354 | isorhamnetin | *CALML3* |
| MOL000354 | isorhamnetin | *PYGM* |
| MOL000354 | isorhamnetin | *PPARD* |
| MOL000354 | isorhamnetin | *CHEK1* |
| MOL000354 | isorhamnetin | *AKR1B1* |
| MOL000354 | isorhamnetin | *NCOA1* |
| MOL000354 | isorhamnetin | *F7* |
| MOL000354 | isorhamnetin | *COLQ* |
| MOL000354 | isorhamnetin | *GABRA1* |
| MOL000354 | isorhamnetin | *MAOB* |
| MOL000354 | isorhamnetin | *GRIA2* |
| MOL000354 | isorhamnetin | *RELA* |
| MOL000354 | isorhamnetin | *NCF1* |
| MOL000354 | isorhamnetin | *OLR1* |
| MOL000358 | beta-sitosterol | *PGR* |
| MOL000358 | beta-sitosterol | *NCOA2* |
| MOL000358 | beta-sitosterol | *PTGS1* |
| MOL000358 | beta-sitosterol | *PTGS2* |
| MOL000358 | beta-sitosterol | *HSP90AA2P* |
| MOL000358 | beta-sitosterol | *KCNH2* |
| MOL000358 | beta-sitosterol | *DRD1* |
| MOL000358 | beta-sitosterol | *CHRM3* |
| MOL000358 | beta-sitosterol | *CHRM1* |
| MOL000358 | beta-sitosterol | *SCN5A* |
| MOL000358 | beta-sitosterol | *CHRM4* |
| MOL000358 | beta-sitosterol | *ADRA1A* |
| MOL000358 | beta-sitosterol | *CHRM2* |
| MOL000358 | beta-sitosterol | *ADRA1B* |
| MOL000358 | beta-sitosterol | *ADRB2* |
| MOL000358 | beta-sitosterol | *CHRNA2* |
| MOL000358 | beta-sitosterol | *SLC6A4* |
| MOL000358 | beta-sitosterol | *OPRM1* |
| MOL000358 | beta-sitosterol | *GABRA1* |
| MOL000358 | beta-sitosterol | *BCL2* |
| MOL000358 | beta-sitosterol | *BAX* |
| MOL000358 | beta-sitosterol | *CASP9* |
| MOL000358 | beta-sitosterol | *JUN* |
| MOL000358 | beta-sitosterol | *CASP3* |
| MOL000358 | beta-sitosterol | *CASP8* |
| MOL000358 | beta-sitosterol | *PRKCA* |
| MOL000358 | beta-sitosterol | *PON1* |
| MOL000358 | beta-sitosterol | *MAP2* |
| MOL000359 | sitosterol | *PGR* |
| MOL000359 | sitosterol | *NCOA2* |
| MOL000359 | sitosterol | *NR3C2* |
| MOL003943 | Rutalinidine | *PTGS1* |
| MOL003943 | Rutalinidine | *DRD1* |
| MOL003943 | Rutalinidine | *CHRM3* |
| MOL003943 | Rutalinidine | *CHRM1* |
| MOL003943 | Rutalinidine | *ESR1* |
| MOL003943 | Rutalinidine | *AR* |
| MOL003943 | Rutalinidine | *SCN5A* |
| MOL003943 | Rutalinidine | *PTGS2* |
| MOL003943 | Rutalinidine | *COLQ* |
| MOL003943 | Rutalinidine | *ADRB2* |
| MOL003943 | Rutalinidine | *OPRM1* |
| MOL003943 | Rutalinidine | *GABRA1* |
| MOL003943 | Rutalinidine | *HSP90AA2P* |
| MOL003943 | Rutalinidine | *DPEP1* |
| MOL003947 | 1-methyl-2-[(Z)-pentadec-10-enyl]-4-quinolone | *CHRM3* |
| MOL003947 | 1-methyl-2-[(Z)-pentadec-10-enyl]-4-quinolone | *KCNH2* |
| MOL003947 | 1-methyl-2-[(Z)-pentadec-10-enyl]-4-quinolone | *AR* |
| MOL003947 | 1-methyl-2-[(Z)-pentadec-10-enyl]-4-quinolone | *ADRB1* |
| MOL003947 | 1-methyl-2-[(Z)-pentadec-10-enyl]-4-quinolone | *SCN5A* |
| MOL003947 | 1-methyl-2-[(Z)-pentadec-10-enyl]-4-quinolone | *SLC6A2* |
| MOL003947 | 1-methyl-2-[(Z)-pentadec-10-enyl]-4-quinolone | *ADRA1B* |
| MOL003947 | 1-methyl-2-[(Z)-pentadec-10-enyl]-4-quinolone | *ADRB2* |
| MOL003956 | dihydrorutaecarpine | *PTGS1* |
| MOL003956 | dihydrorutaecarpine | *CHRM3* |
| MOL003956 | dihydrorutaecarpine | *CHRM1* |
| MOL003956 | dihydrorutaecarpine | *AR* |
| MOL003956 | dihydrorutaecarpine | *SCN5A* |
| MOL003956 | dihydrorutaecarpine | *PTGS2* |
| MOL003956 | dihydrorutaecarpine | *HTR3A* |
| MOL003956 | dihydrorutaecarpine | *CA2* |
| MOL003956 | dihydrorutaecarpine | *RXRA* |
| MOL003956 | dihydrorutaecarpine | *ADRB2* |
| MOL003956 | dihydrorutaecarpine | *HSP90AA2P* |
| MOL003956 | dihydrorutaecarpine | *PRSS1* |
| MOL003957 | 1-methyl-2-pentadecyl-4-quinolone | *CHRM3* |
| MOL003957 | 1-methyl-2-pentadecyl-4-quinolone | *KCNH2* |
| MOL003957 | 1-methyl-2-pentadecyl-4-quinolone | *CHRM1* |
| MOL003957 | 1-methyl-2-pentadecyl-4-quinolone | *AR* |
| MOL003957 | 1-methyl-2-pentadecyl-4-quinolone | *ADRB1* |
| MOL003957 | 1-methyl-2-pentadecyl-4-quinolone | *SCN5A* |
| MOL003957 | 1-methyl-2-pentadecyl-4-quinolone | *ADRA2C* |
| MOL003957 | 1-methyl-2-pentadecyl-4-quinolone | *SLC6A2* |
| MOL003957 | 1-methyl-2-pentadecyl-4-quinolone | *ADRA1B* |
| MOL003957 | 1-methyl-2-pentadecyl-4-quinolone | *ADRB2* |
| MOL003957 | 1-methyl-2-pentadecyl-4-quinolone | *CCNA2* |
| MOL003957 | 1-methyl-2-pentadecyl-4-quinolone | *CALML3* |
| MOL003958 | Evodiamine | *PTGS1* |
| MOL003958 | Evodiamine | *CHRM3* |
| MOL003958 | Evodiamine | *KCNH2* |
| MOL003958 | Evodiamine | *AR* |
| MOL003958 | Evodiamine | *SCN5A* |
| MOL003958 | Evodiamine | *CHRM5* |
| MOL003958 | Evodiamine | *PTGS2* |
| MOL003958 | Evodiamine | *HTR3A* |
| MOL003958 | Evodiamine | *CA2* |
| MOL003958 | Evodiamine | *F7* |
| MOL003958 | Evodiamine | *RXRA* |
| MOL003958 | Evodiamine | *GABRA1* |
| MOL003958 | Evodiamine | *HSP90AA2P* |
| MOL003958 | Evodiamine | *PRSS1* |
| MOL003958 | Evodiamine | *CHRM1* |
| MOL003958 | Evodiamine | *ADRB2* |
| MOL003958 | Evodiamine | *NCOA1* |
| MOL003960 | 1-(5,7,8-trimethoxy-2,2-dimethylchromen-6-yl)ethanone | *PTGS1* |
| MOL003960 | 1-(5,7,8-trimethoxy-2,2-dimethylchromen-6-yl)ethanone | *CHRM3* |
| MOL003960 | 1-(5,7,8-trimethoxy-2,2-dimethylchromen-6-yl)ethanone | *CHRM1* |
| MOL003960 | 1-(5,7,8-trimethoxy-2,2-dimethylchromen-6-yl)ethanone | *SCN5A* |
| MOL003960 | 1-(5,7,8-trimethoxy-2,2-dimethylchromen-6-yl)ethanone | *PTGS2* |
| MOL003960 | 1-(5,7,8-trimethoxy-2,2-dimethylchromen-6-yl)ethanone | *RXRA* |
| MOL003960 | 1-(5,7,8-trimethoxy-2,2-dimethylchromen-6-yl)ethanone | *ADRA1B* |
| MOL003960 | 1-(5,7,8-trimethoxy-2,2-dimethylchromen-6-yl)ethanone | *SLC6A3* |
| MOL003960 | 1-(5,7,8-trimethoxy-2,2-dimethylchromen-6-yl)ethanone | *SLC6A4* |
| MOL003960 | 1-(5,7,8-trimethoxy-2,2-dimethylchromen-6-yl)ethanone | *OPRM1* |
| MOL003960 | 1-(5,7,8-trimethoxy-2,2-dimethylchromen-6-yl)ethanone | *HSP90AA2P* |
| MOL003963 | hydroxyevodiamine | *PTGS1* |
| MOL003963 | hydroxyevodiamine | *AR* |
| MOL003963 | hydroxyevodiamine | *SCN5A* |
| MOL003963 | hydroxyevodiamine | *PTGS2* |
| MOL003963 | hydroxyevodiamine | *CA2* |
| MOL003963 | hydroxyevodiamine | *F7* |
| MOL003963 | hydroxyevodiamine | *KDR* |
| MOL003963 | hydroxyevodiamine | *RXRA* |
| MOL003963 | hydroxyevodiamine | *HSP90AA2P* |
| MOL003963 | hydroxyevodiamine | *PRSS1* |
| MOL003963 | hydroxyevodiamine | *NCOA2* |
| MOL003964 | 1-methyl-2-undecyl-4-quinolone | *CHRM3* |
| MOL003964 | 1-methyl-2-undecyl-4-quinolone | *CHRM1* |
| MOL003964 | 1-methyl-2-undecyl-4-quinolone | *AR* |
| MOL003964 | 1-methyl-2-undecyl-4-quinolone | *ADRB1* |
| MOL003964 | 1-methyl-2-undecyl-4-quinolone | *SCN5A* |
| MOL003964 | 1-methyl-2-undecyl-4-quinolone | *ADRA2C* |
| MOL003964 | 1-methyl-2-undecyl-4-quinolone | *SLC6A2* |
| MOL003964 | 1-methyl-2-undecyl-4-quinolone | *ADRA1B* |
| MOL003964 | 1-methyl-2-undecyl-4-quinolone | *SLC6A3* |
| MOL003964 | 1-methyl-2-undecyl-4-quinolone | *ADRB2* |
| MOL003964 | 1-methyl-2-undecyl-4-quinolone | *SLC6A4* |
| MOL003964 | 1-methyl-2-undecyl-4-quinolone | *CCNA2* |
| MOL003964 | 1-methyl-2-undecyl-4-quinolone | *CALML3* |
| MOL003972 | 1-methyl-2-nonyl-4-quinolone | *CHRM3* |
| MOL003972 | 1-methyl-2-nonyl-4-quinolone | *CHRM1* |
| MOL003972 | 1-methyl-2-nonyl-4-quinolone | *AR* |
| MOL003972 | 1-methyl-2-nonyl-4-quinolone | *ADRB1* |
| MOL003972 | 1-methyl-2-nonyl-4-quinolone | *SCN5A* |
| MOL003972 | 1-methyl-2-nonyl-4-quinolone | *PTGS2* |
| MOL003972 | 1-methyl-2-nonyl-4-quinolone | *ADRA2C* |
| MOL003972 | 1-methyl-2-nonyl-4-quinolone | *CHRNA10* |
| MOL003972 | 1-methyl-2-nonyl-4-quinolone | *RXRA* |
| MOL003972 | 1-methyl-2-nonyl-4-quinolone | *SLC6A2* |
| MOL003972 | 1-methyl-2-nonyl-4-quinolone | *CHRM2* |
| MOL003972 | 1-methyl-2-nonyl-4-quinolone | *ADRA2B* |
| MOL003972 | 1-methyl-2-nonyl-4-quinolone | *ADRA1B* |
| MOL003972 | 1-methyl-2-nonyl-4-quinolone | *SLC6A3* |
| MOL003972 | 1-methyl-2-nonyl-4-quinolone | *ADRB2* |
| MOL003972 | 1-methyl-2-nonyl-4-quinolone | *ADRA1D* |
| MOL003972 | 1-methyl-2-nonyl-4-quinolone | *SLC6A4* |
| MOL003972 | 1-methyl-2-nonyl-4-quinolone | *MAOB* |
| MOL003972 | 1-methyl-2-nonyl-4-quinolone | *CCNA2* |
| MOL003974 | Evocarpine | *CHRM3* |
| MOL003974 | Evocarpine | *KCNH2* |
| MOL003974 | Evocarpine | *CHRM1* |
| MOL003974 | Evocarpine | *AR* |
| MOL003974 | Evocarpine | *SCN5A* |
| MOL003974 | Evocarpine | *ADRA2C* |
| MOL003974 | Evocarpine | *SLC6A2* |
| MOL003974 | Evocarpine | *ADRA1B* |
| MOL003974 | Evocarpine | *SLC6A3* |
| MOL003974 | Evocarpine | *ADRB2* |
| MOL003974 | Evocarpine | *SLC6A4* |
| MOL003994 | 24-methyl-31-norlanost-9(11)-enol | *PGR* |
| MOL004004 | 6-OH-Luteolin | *PTGS1* |
| MOL004004 | 6-OH-Luteolin | *AR* |
| MOL004004 | 6-OH-Luteolin | *PTGS2* |
| MOL004004 | 6-OH-Luteolin | *HSP90AA2P* |
| MOL004004 | 6-OH-Luteolin | *NCOA2* |
| MOL004014 | Evodiamide | *PTGS1* |
| MOL004014 | Evodiamide | *KCNH2* |
| MOL004014 | Evodiamide | *CHRM1* |
| MOL004014 | Evodiamide | *AR* |
| MOL004014 | Evodiamide | *SCN5A* |
| MOL004014 | Evodiamide | *PTGS2* |
| MOL004014 | Evodiamide | *RXRA* |
| MOL004014 | Evodiamide | *ADRA1B* |
| MOL004014 | Evodiamide | *ADRB2* |
| MOL004014 | Evodiamide | *GABRA1* |
| MOL004014 | Evodiamide | *BACE2* |
| MOL004014 | Evodiamide | *HSP90AA2P* |
| MOL004014 | Evodiamide | *NCOA2* |
| MOL004018 | Goshuyuamide I | *PTGS1* |
| MOL004018 | Goshuyuamide I | *CHRM3* |
| MOL004018 | Goshuyuamide I | *KCNH2* |
| MOL004018 | Goshuyuamide I | *CHRM1* |
| MOL004018 | Goshuyuamide I | *AR* |
| MOL004018 | Goshuyuamide I | *ADRB1* |
| MOL004018 | Goshuyuamide I | *SCN5A* |
| MOL004018 | Goshuyuamide I | *CHRM5* |
| MOL004018 | Goshuyuamide I | *PTGS2* |
| MOL004018 | Goshuyuamide I | *F7* |
| MOL004018 | Goshuyuamide I | *RXRA* |
| MOL004018 | Goshuyuamide I | *ADRA1B* |
| MOL004018 | Goshuyuamide I | *ADRB2* |
| MOL004018 | Goshuyuamide I | *ADRA1D* |
| MOL004018 | Goshuyuamide I | *GABRA1* |
| MOL004018 | Goshuyuamide I | *BACE2* |
| MOL004018 | Goshuyuamide I | *HSP90AA2P* |
| MOL004018 | Goshuyuamide I | *NCOA2* |
| MOL004019 | GoshuyuamideII | *PTGS1* |
| MOL004019 | GoshuyuamideII | *CHRM3* |
| MOL004019 | GoshuyuamideII | *KCNH2* |
| MOL004019 | GoshuyuamideII | *CHRM1* |
| MOL004019 | GoshuyuamideII | *SCN5A* |
| MOL004019 | GoshuyuamideII | *CHRM5* |
| MOL004019 | GoshuyuamideII | *PTGS2* |
| MOL004019 | GoshuyuamideII | *F7* |
| MOL004019 | GoshuyuamideII | *CHRM4* |
| MOL004019 | GoshuyuamideII | *ADRA1B* |
| MOL004019 | GoshuyuamideII | *ADRB2* |
| MOL004019 | GoshuyuamideII | *ADRA1D* |
| MOL004019 | GoshuyuamideII | *HSP90AA2P* |
| MOL004019 | GoshuyuamideII | *CALML3* |
| MOL004020 | gossypetin | *AR* |
| MOL004020 | gossypetin | *PPARG* |
| MOL004020 | gossypetin | *PTGS2* |
| MOL004020 | gossypetin | *HSP90AA2P* |
| MOL004020 | gossypetin | *DPP4* |
| MOL004020 | gossypetin | *CYP19A1* |
| MOL004020 | gossypetin | *PARP1* |
| MOL004021 | Gravacridoneshlirine | *NOS2* |
| MOL004021 | Gravacridoneshlirine | *PTGS1* |
| MOL004021 | Gravacridoneshlirine | *AR* |
| MOL004021 | Gravacridoneshlirine | *PTGS2* |
| MOL004021 | Gravacridoneshlirine | *CALML3* |
| MOL004025 | N-(2-Methylaminobenzoyl)tryptamine | *PTGS1* |
| MOL004025 | N-(2-Methylaminobenzoyl)tryptamine | *CHRM1* |
| MOL004025 | N-(2-Methylaminobenzoyl)tryptamine | *AR* |
| MOL004025 | N-(2-Methylaminobenzoyl)tryptamine | *ADRB1* |
| MOL004025 | N-(2-Methylaminobenzoyl)tryptamine | *SCN5A* |
| MOL004025 | N-(2-Methylaminobenzoyl)tryptamine | *PTGS2* |
| MOL004025 | N-(2-Methylaminobenzoyl)tryptamine | *RXRA* |
| MOL004025 | N-(2-Methylaminobenzoyl)tryptamine | *ADRB2* |
| MOL004025 | N-(2-Methylaminobenzoyl)tryptamine | *BACE2* |
| MOL000098 | quercetin | *PTGS1* |
| MOL000098 | quercetin | *AR* |
| MOL000098 | quercetin | *PPARG* |
| MOL000098 | quercetin | *PTGS2* |
| MOL000098 | quercetin | *HSP90AA2P* |
| MOL000098 | quercetin | *NCOA2* |
| MOL000098 | quercetin | *DPP4* |
| MOL000098 | quercetin | *AKR1B1* |
| MOL000098 | quercetin | *PRSS1* |
| MOL000098 | quercetin | *KCNH2* |
| MOL000098 | quercetin | *SCN5A* |
| MOL000098 | quercetin | *ADRB2* |
| MOL000098 | quercetin | *MMP3* |
| MOL000098 | quercetin | *F7* |
| MOL000098 | quercetin | *RXRA* |
| MOL000098 | quercetin | *COLQ* |
| MOL000098 | quercetin | *GABRA1* |
| MOL000098 | quercetin | *MAOB* |
| MOL000098 | quercetin | *RELA* |
| MOL000098 | quercetin | *EGFR* |
| MOL000098 | quercetin | *AKT1* |
| MOL000098 | quercetin | *VEGFA* |
| MOL000098 | quercetin | *CCND1* |
| MOL000098 | quercetin | *BCL2* |
| MOL000098 | quercetin | *BCL2L1* |
| MOL000098 | quercetin | *FOS* |
| MOL000098 | quercetin | *CDKN1A* |
| MOL000098 | quercetin | *EIF6* |
| MOL000098 | quercetin | *BAX* |
| MOL000098 | quercetin | *CASP9* |
| MOL000098 | quercetin | *PLAU* |
| MOL000098 | quercetin | *MMP2* |
| MOL000098 | quercetin | *MMP9* |
| MOL000098 | quercetin | *MAPK1* |
| MOL000098 | quercetin | *IL10RB* |
| MOL000098 | quercetin | *EGF* |
| MOL000098 | quercetin | *RB1* |
| MOL000098 | quercetin | *TNF* |
| MOL000098 | quercetin | *JUN* |
| MOL000098 | quercetin | *IL6ST* |
| MOL000098 | quercetin | *AHSA1* |
| MOL000098 | quercetin | *CASP3* |
| MOL000098 | quercetin | *TP53* |
| MOL000098 | quercetin | *ELK1* |
| MOL000098 | quercetin | *NFKBIA* |
| MOL000098 | quercetin | *POR* |
| MOL000098 | quercetin | *ODC1* |
| MOL000098 | quercetin | *CASP8* |
| MOL000098 | quercetin | *TOP1* |
| MOL000098 | quercetin | *RAF1* |
| MOL000098 | quercetin | *SOD1* |
| MOL000098 | quercetin | *PRKCA* |
| MOL000098 | quercetin | *MMP1* |
| MOL000098 | quercetin | *HIF1A* |
| MOL000098 | quercetin | *STAT1* |
| MOL000098 | quercetin | *RUNX1T1* |
| MOL000098 | quercetin | *CDK1* |
| MOL000098 | quercetin | *HSPA5* |
| MOL000098 | quercetin | *ERBB2* |
| MOL000098 | quercetin | *PPARG* |
| MOL000098 | quercetin | *ACACA* |
| MOL000098 | quercetin | *HMOX1* |
| MOL000098 | quercetin | *CYP3A4* |
| MOL000098 | quercetin | *CYP1A2* |
| MOL000098 | quercetin | *CAV1* |
| MOL000098 | quercetin | *MYC* |
| MOL000098 | quercetin | *F3* |
| MOL000098 | quercetin | *GJA1* |
| MOL000098 | quercetin | *CYP1A1* |
| MOL000098 | quercetin | *ICAM1* |
| MOL000098 | quercetin | *IL1B* |
| MOL000098 | quercetin | *CCL2* |
| MOL000098 | quercetin | *SELE* |
| MOL000098 | quercetin | *VCAM1* |
| MOL000098 | quercetin | *PTGER3* |
| MOL000098 | quercetin | *CXCL8* |
| MOL000098 | quercetin | *PRKCB* |
| MOL000098 | quercetin | *BIRC5* |
| MOL000098 | quercetin | *DUOX2* |
| MOL000098 | quercetin | *NOS3* |
| MOL000098 | quercetin | *HSPB1* |
| MOL000098 | quercetin | *SULT1E1* |
| MOL000098 | quercetin | *IL2RA* |
| MOL000098 | quercetin | *NR1I2* |
| MOL000098 | quercetin | *CYP1B1* |
| MOL000098 | quercetin | *CCNB1* |
| MOL000098 | quercetin | *PLAT* |
| MOL000098 | quercetin | *THBD* |
| MOL000098 | quercetin | *SERPINE1* |
| MOL000098 | quercetin | *COL1A1* |
| MOL000098 | quercetin | *IFNG* |
| MOL000098 | quercetin | *ALOX5* |
| MOL000098 | quercetin | *IL1A* |
| MOL000098 | quercetin | *MPO* |
| MOL000098 | quercetin | *TOP2A* |
| MOL000098 | quercetin | *NCF1* |
| MOL000098 | quercetin | *ABCG2* |
| MOL000098 | quercetin | *HAS2* |
| MOL000098 | quercetin | *GSTP1* |
| MOL000098 | quercetin | *NFE2L2* |
| MOL000098 | quercetin | *NQO1* |
| MOL000098 | quercetin | *PARP1* |
| MOL000098 | quercetin | *AHR* |
| MOL000098 | quercetin | *PSMD3* |
| MOL000098 | quercetin | *SLC2A4* |
| MOL000098 | quercetin | *COL3A1* |
| MOL000098 | quercetin | *CXCL11* |
| MOL000098 | quercetin | *CXCL2* |
| MOL000098 | quercetin | *DCAF5* |
| MOL000098 | quercetin | *NR1I3* |
| MOL000098 | quercetin | *CHEK2* |
| MOL000098 | quercetin | *INSRR* |
| MOL000098 | quercetin | *CLDN4* |
| MOL000098 | quercetin | *PPARA* |
| MOL000098 | quercetin | *PPARD* |
| MOL000098 | quercetin | *HSF1* |
| MOL000098 | quercetin | *CXCL10* |
| MOL000098 | quercetin | *CHUK* |
| MOL000098 | quercetin | *SPP1* |
| MOL000098 | quercetin | *RUNX2* |
| MOL000098 | quercetin | *RASSF1* |
| MOL000098 | quercetin | *E2F1* |
| MOL000098 | quercetin | *E2F2* |
| MOL000098 | quercetin | *ACP3* |
| MOL000098 | quercetin | *CTSD* |
| MOL000098 | quercetin | *IGFBP3* |
| MOL000098 | quercetin | *IGF2* |
| MOL000098 | quercetin | *CD40LG* |
| MOL000098 | quercetin | *IRF1* |
| MOL000098 | quercetin | *ERBB3* |
| MOL000098 | quercetin | *PON1* |
| MOL000098 | quercetin | *DIO1* |
| MOL000098 | quercetin | *PCOLCE* |
| MOL000098 | quercetin | *NPEPPS* |
| MOL000098 | quercetin | *HK2* |
| MOL000098 | quercetin | *RASA1* |
| MOL000098 | quercetin | *GSTM1* |
| MOL000098 | quercetin | *GSTM2* |
